# Supplementary material for: Evaluation of left ventricular ejection fraction by a new automatic tool on a pocket ultrasound device: Concordance study with cardiac magnetic resonance imaging
Source: PLoS One. 2024 Aug 12;19(8):e0308580. doi: 10.1371/journal.pone.0308580 (PMC11318925; doi:10.1371/journal.pone.0308580)
Supplement: S1 File — (PDF) [file pone.0308580.s001.pdf]

ic1	ic2	ic3	nic1	nic2	nic3	nic4	nic5	nic6	nic7	nic8	med_hist	med_hist_9	med_hist_6	med_hist_3	med_hist_1	med_hist_2	med_hist_4	med_hist_5	med_hist_7	med_hist_8	med_hist_99	previous_LVEF_YN	previsous_LVEF	sbp	dbp	hr	af	spo2	rr	glasg	indic_mri	pud_qual	pud_visual_yn	pud_visual_lvef	pud_auto_yn	pud_auto_lvef	cu_qual	cu_visual_yn	cu_visual_lvef	cu_auto_yn	cu_auto_lvef	mapse_yn	mapse	sa_yn	sa	lvef_mri	diag_mri	pud_auto_lvef_alt	pud_visual_lvef_alt	lvef_mri_alt	pud_auto_lvef_alts	pud_visual_lvef_alts	lvef_mri_alts	mapse_inf7	mapse_inf10	sa_inf6	sa_inf9	lvef_mri_cl	pud_auto_lvef_cl	pud_visual_lvef_cl	mapse_cl	sa_cl
1	1	1	0	0	0	0	0	0	0	0	99	0	0	0	0	0	0	0	0	0	1	1	50	95	51	76	0	100	18	15	1	4	1	50	1	45	5	1	50	1	43	1	12	1	14,9	47	3	1	0	1	0	0	0	0	0	0	0	2	2	3	3	3
1	1	1	0	0	0	0	0	0	0	0	6-1-4	0	1	0	1	0	1	0	0	0	0	1	50	117	66	78	0	94	15	15	2	3	1	35	1	40	3	1	30	1	35	1	10	1	8,4	43	3	1	1	1	0	0	0	0	1	0	1	2	2	2	2	2
1	1	1	0	0	0	0	0	0	0	0	5	0	0	0	0	0	0	1	0	0	0	0		135	68	65	0	97	16	15	1	4	1	60	1	49	5	1	65	1	45	1	9	1	8,4	66	2	1	0	0	0	0	0	0	1	0	1	3	2	3	2	2
1	1	1	0	0	0	0	0	0	0	0	99	0	0	0	0	0	0	0	0	0	1	1	65	138	69	72	0	98	18	15	1	3	1	50	1	46	3	1	50	1	34	1	13	1	11,6	57	1	1	0	0	0	0	0	0	0	0	0	3	2	3	3	3
1	1	1	0	0	0	0	0	0	0	0	6-5	0	1	0	0	0	0	1	0	0	0	1	50	146	85	63	0	95	20	15	2	4	1	50	1	30	1	0		0		0		0		45	3	1	0	1	0	0	0					2	2	3		
1	1	1	0	0	0	0	0	0	0	0	99	0	0	0	0	0	0	0	0	0	1	0		127	63	68	0	99	15	15	1	3	1	50	1	37	3	1	55	0		0		0		68	1	1	0	0	0	0	0					3	2	3		
1	1	1	0	0	0	0	0	0	0	0	99	0	0	0	0	0	0	0	0	0	1	0		135	65	79	0	97	20	15	1	3	1	50	1	41	4	1	55	0		0		0		51	2	1	0	0	0	0	0					3	2	3		
1	1	1	0	0	0	0	0	0	0	0	9-6-3-2-5	1	1	1	0	1	0	1	0	0	0	1	34	149	82	58	1	94	16	15	1	3	1	20	1	23	4	1	20	0		1	4	0		19	3	1	1	1	1	1	1	1	1			1	1	1	1	
1	1	1	0	0	0	0	0	0	0	0	4	0	0	0	0	0	1	0	0	0	0	0		139	58	71	0	97	17	15	1	5	1	55	1	49	1	0		0		0		0		60	1	1	0	0	0	0	0					3	2	3		
1	1	1	0	0	0	0	0	0	0	0	9-6-3-1-2-5-8	1	1	1	1	1	0	1	0	1	0	1	45	145	89	85	1	94	15	15	1	5	1	45	1	39	1	0		0		0		0		54	2	1	1	0	0	0	0					3	2	2		
1	1	1	0	0	0	0	0	0	0	0	9-3-1-2-4-5-7	1	0	1	1	1	1	1	1	0	0	1	33	151	72	80	0	93	16	15	1	3	1	15	0		4	1	20	0		1	6	1	5	29	3		1	1		1	1	1	1	1	1	1		1	1	1
1	1	1	0	0	0	0	0	0	0	0	99	0	0	0	0	0	0	0	0	0	1	1	61	118	62	54	0	100	14	15	1	5	1	55	1	45	1	0		0		0		0		49	2	1	0	1	0	0	0					2	2	3		
1	1	1	0	0	0	0	0	0	0	0	1-5	0	0	0	1	0	0	1	0	0	0	0		141	58	78	0	95	15	15	2	4	1	55	1	46	1	0		0		0		0		38	2	1	0	1	0	0	0					2	2	3		
1	1	1	0	0	0	0	0	0	0	0	1-7	0	0	0	1	0	0	0	1	0	0	1	60	135	69	70	0	96	19	15	1	3	1	50	1	39	3	1	50	0		1	8	1	9	44	2	1	0	1	0	0	0	0	1	0	0	2	2	3	2	3
1	1	1	0	0	0	0	0	0	0	0	3-1-2-5	0	0	1	1	1	0	1	0	0	0	0		142	66	71	0	98	15	15	1	3	1	55	0		3	1	55	0		1	8	0		55	2		0	0		0	0	0	1			3		3	2	
1	1	1	0	0	0	0	0	0	0	0	1-2-5	0	0	0	1	1	0	1	0	0	0	1	58	139	66	58	0	94	14	15	2	5	1	55	1	60	5	1	55	0		1	9	1	7	54	3	0	0	0	0	0	0	0	1	0	1	3	3	3	2	2
1	1	1	0	0	0	0	0	0	0	0	9-2	1	0	0	0	1	0	0	0	0	0	1	42	135	60	77	0	98	20	15	1	4	1	45	1	36	5	1	40	0		1	7	1	8	45	2	1	1	1	0	0	0	0	1	0	1	2	2	2	2	2
1	1	1	0	0	0	0	0	0	0	0	9-1-5	1	0	0	1	0	0	1	0	0	0	0		146	78	78	0	95	16	15	1	4	1	50	1	34	1	0		0		0		0		53	3	1	0	0	0	0	0					3	2	3		
1	1	1	0	0	0	0	0	0	0	0	5	0	0	0	0	0	0	1	0	0	0	0		116	58	59	0	100	16	15	1	4	1	55	1	30	5	1	55	0		1	13	1	9	63	1	1	0	0	0	0	0	0	0	0	0	3	2	3	3	3
1	1	1	0	0	0	0	0	0	0	0	9-1	1	0	0	1	0	0	0	0	0	0	1	21	148	82	82	0	97	16	15	1	5	1	30	1	24	5	1	35	0		1	2	1	3	32	3	1	1	1	1	0	0	1	1	1	1	2	1	2	1	1
1	1	1	0	0	0	0	0	0	0	0	9-6-1-2-5	1	1	0	1	1	0	1	0	0	0	1	34	120	70	70	0	100	15	15	1	5	1	55	1	44	5	1	60	0		1	18	1	9	49	3	1	0	1	0	0	0	0	0	0	0	2	2	3	3	3
1	1	1	0	0	0	0	0	0	0	0	99	0	0	0	0	0	0	0	0	0	1	1	45	120	70	74	0	100	15	15	1	5	1	50	1	35	1	0		0		0		0		54	1	1	0	0	0	0	0					3	2	3		
1	1	1	0	0	0	0	0	0	0	0	2-4-5	0	0	0	0	1	1	1	0	0	0	0		130	80	75	0	99	16	15	1	4	1	50	1	24	1	0		0		0		0		53	1	1	0	0	1	0	0					3	1	3		
1	1	1	0	0	0	0	0	0	0	0	3-1-2	0	0	1	1	1	0	0	0	0	0	1	60	120	70	80	0	98	20	15	1	4	1	50	1	36	1	0		0		0		0		37	3	1	0	1	0	0	0					2	2	3		
1	1	1	0	0	0	0	0	0	0	0	9-1-2-4-5	1	0	0	1	1	1	1	0	0	0	0		143	59	81	0	97	16	15	1	4	1	40	1	42	4	1	45	0		1	14	1	9	46	2	1	1	1	0	0	0	0	0	0	0	2	2	2	3	3
1	1	1	0	0	0	0	0	0	0	0	5	0	0	0	0	0	0	1	0	0	0	0		123	80	62	0	97	14	15	1	3	1	50	1	40	1	0		0		0		0		57	2	1	0	0	0	0	0					3	2	3		
1	1	1	0	0	0	0	0	0	0	0	6-5	0	1	0	0	0	0	1	0	0	0	0		142	76	73	0	96	17	15	2	5	1	50	1	48	5	1	50	0		1	13	1	8	58	2	1	0	0	0	0	0	0	0	0	1	3	2	3	3	2
1	1	1	0	0	0	0	0	0	0	0	1	0	0	0	1	0	0	0	0	0	0	0		149	85	72	0	97	16	15	1	3	1	55	0		1	0		0		0		0		56	2		0	0		0	0					3		3		
1	1	1	0	0	0	0	0	0	0	0	4	0	0	0	0	0	1	0	0	0	0	0		122	68	59	0	100	14	15	1	4	1	55	1	36	4	1	50	0		1	11	1	12	51	3	1	0	0	0	0	0	0	0	0	0	3	2	3	3	3
1	1	1	0	0	0	0	0	0	0	0	1-2-7	0	0	0	1	1	0	0	1	0	0	1	55	143	72	65	0	96	14	15	2	4	1	50	1	31	4	1	50	0		1	13	1	11	49	3	1	0	1	0	0	0	0	0	0	0	2	2	3	3	3
1	1	1	0	0	0	0	0	0	0	0	5	0	0	0	0	0	0	1	0	0	0	1	57	122	76	58	0	100	15	15	1	3	1	60	0		1	1	60	0		1	12	1	8	55	2		0	0		0	0	0	0	0	1	3		3	3	2
1	1	1	0	0	0	0	0	0	0	0	1-2	0	0	0	1	1	0	0	0	0	0	0		132	75	80	0	99	16	15	1	3	1	45	1	33	4	1	30	0		0		0		62	1	1	1	0	0	0	0					3	2	2		
1	1	1	0	0	0	0	0	0	0	0	2	0	0	0	0	1	0	0	0	0	0	0		127	59	72	0	98	18	15	1	2	1	50	1	38	4	1	50	0		0		0		59	2	1	0	0	0	0	0					3	2	3		
1	1	1	0	0	0	0	0	0	0	0	3-1-2-4	0	0	1	1	1	1	0	0	0	0	1	60	148	72	80	0	96	15	15	1	5	1	55	1	59	5	1	55	0		1	9	1	7	60	2	0	0	0	0	0	0	0	1	0	1	3	3	3	2	2
1	1	1	0	0	0	0	0	0	0	0	1	0	0	0	1	0	0	0	0	0	0	0		119	62	64	0	97	16	15	1	4	1	55	1	29	1	0		0		0		0		60	3	1	0	0	1	0	0					3	1	3		
1	1	1	0	0	0	0	0	0	0	0	3-1	0	0	1	1	0	0	0	0	0	0	1	65	133	75	80	0	99	18	15	1	2	1	50	1	47	3	1	45	0		0		0		67	3	1	0	0	0	0	0					3	2	3		
1	1	1	0	0	0	0	0	0	0	0	99	0	0	0	0	0	0	0	0	0	1	1	40	140	70	72	0	100	12	15	1	4	1	55	1	60	4	1	40	0		1	10	1	7	42	3	0	0	1	0	0	0	0	1	0	1	2	3	3	2	2
1	1	1	0	0	0	0	0	0	0	0	99	0	0	0	0	0	0	0	0	0	1	0		150	90	65	0	100	10	15	1	3	1	50	1	43	4	1	50	0		1	13	1	14	75	3	1	0	0	0	0	0	0	0	0	0	3	2	3	3	3
1	1	1	0	0	0	0	0	0	0	0	5	0	0	0	0	0	0	1	0	0	0	1	20	91	60	101	0	97	14	15	1	4	1	20	1	35	1	0		0		0		0		21	3	1	1	1	0	1	1					1	2	1		
1	1	1	0	0	0	0	0	0	0	0	5	0	0	0	0	0	0	1	0	0	0	1	65	128	80	75	0	100	13	15	1	5	1	55	1	38	5	1	55	0		1	17	1	12	59	3	1	0	0	0	0	0	0	0	0	0	3	2	3	3	3
1	1	1	0	0	0	0	0	0	0	0	4-5	0	0	0	0	0	1	1	0	0	0	1	49	124	79	74	0	99	14	15	1	4	1	60	1	39	1	0		0		0		0		47	3	1	0	1	0	0	0					2	2	3		
1	1	1	0	0	0	0	0	0	0	0	3-2	0	0	1	0	1	0	0	0	0	0	1	60	138	82	63	0	100	13	15	1	4	1	40	1	26	4	1	35	0		1	10	1	6	46	3	1	1	1	1	0	0	0	1	0	1	2	1	2	2	2
1	1	1	0	0	0	0	0	0	0	0	99	0	0	0	0	0	0	0	0	0	1	1	50	111	74	60	0	98	17	15	1	4	1	45	1	36	4	1	45	0		1	11	1	7	43	3	1	1	1	0	0	0	0	0	0	1	2	2	2	3	2
1	1	1	0	0	0	0	0	0	0	0	9-1-5-8	1	0	0	1	0	0	1	0	1	0	1	39	140	88	70	1	97	15	15	1	3	1	40	1	37	4	1	40	0		1	11	1	5	48	3	1	1	1	0	0	0	0	0	1	1	2	2	2	3	1
1	1	1	0	0	0	0	0	0	0	0	6-1-2-5	0	1	0	1	1	0	1	0	0	0	1	43	105	92	55	0	97	13	15	2	5	1	45	1	46	1	0		0		0		0		47	3	1	1	1	0	0	0					2	2	2		
1	1	1	0	0	0	0	0	0	0	0	9-6-1-2-4-5-8	1	1	0	1	1	1	1	0	1	0	1	29	126	59	63	0	98	16	15	1	3	1	40	1	35	4	1	40	0		1	9	1	6	30	3	1	1	1	0	0	0	0	1	0	1	2	2	2	2	2
1	1	1	0	0	0	0	0	0	0	0	99	0	0	0	0	0	0	0	0	0	1	1	57	118	54	57	0	100	13	15	1	4	1	55	1	58	1	0		0		0		0		54	2	0	0	0	0	0	0					3	3	3		
1	1	1	0	0	0	0	0	0	0	0	1-4	0	0	0	1	0	1	0	0	0	0	1	20	134	71	53	0	96	17	15	2	3	1	20	0		3	1	25	0		1	8	1	5	36	3		1	1		1	0	0	1	1	1	2		1	2	1
1	1	1	0	0	0	0	0	0	0	0	6-5	0	1	0	0	0	0	1	0	0	0	1	38	121	57	62	0	100	12	15	1	5	1	45	1	35	5	1	45	0		1	12	1	8	41	3	1	1	1	0	0	0	0	0	0	1	2	2	2	3	2
1	1	1	0	0	0	0	0	0	0	0	99	0	0	0	0	0	0	0	0	0	1	0		118	56	66	0	100	14	15	2	4	1	55	1	39	4	1	55	0		1	13	1	7	54	2	1	0	0	0	0	0	0	0	0	1	3	2	3	3	2
1	1	1	0	0	0	0	0	0	0	0	9-1-4-8	1	0	0	1	0	1	0	0	1	0	1	45	137	61	68	1	99	14	15	1	3	1	50	1	54	4	1	50	0		1	10	1	10	56	3	0	0	0	0	0	0	0	1	0	0	3	3	3	2	3
1	1	1	0	0	0	0	0	0	0	0	4	0	0	0	0	0	1	0	0	0	0	0		128	55	54	0	98	15	15	1	3	1	55	0		1	0		0		0		0		60	1		0	0		0	0					3		3		
1	1	1	0	0	0	0	0	0	0	0	3-1-4-8	0	0	1	1	0	1	0	0	1	0	0		140	65	53	1	95	13	15	1	5	1	55	1	50	5	1	55	0		1	14	1	12	51	2	0	0	0	0	0	0	0	0	0	0	3	3	3	3	3
1	1	1	0	0	0	0	0	0	0	0	9-6-3-1-2-8	1	1	1	1	1	0	0	0	1	0	1	40	138	62	55	1	98	13	15	2	4	1	40	1	34	1	0		0		0		0		45	3	1	1	1	0	0	0					2	2	2		
1	1	1	0	0	0	0	0	0	0	0	9-6-2-5-8	1	1	0	0	1	0	1	0	1	0	1	40	125	80	60	0	98	16	15	1	3	1	45	0		3	1	40	0		0		0		30	3		1	1		0	0					2		2		
1	1	1	0	0	0	0	0	0	0	0	6-2	0	1	0	0	1	0	0	0	0	0	1	55	124	69	70	0	100	12	15	1	5	1	55	1	37	5	1	55	0		1	10	1	11	47	2	1	0	1	0	0	0	0	1	0	0	2	2	3	2	3
1	1	1	0	0	0	0	0	0	0	0	2-4-5	0	0	0	0	1	1	1	0	0	0	1	55	118	61	55	0	100	12	15	2	5	1	55	1	49	5	1	55	0		1	17	1	11	55	2	1	0	0	0	0	0	0	0	0	0	3	2	3	3	3
1	1	1	0	0	0	0	0	0	0	0	1-2-5	0	0	0	1	1	0	1	0	0	0	0		125	72	62	0	98	14	15	1	5	1	60	1	60	5	1	60	0		1	18	1	10	56	1	0	0	0	0	0	0	0	0	0	0	3	3	3	3	3
1	1	1	0	0	0	0	0	0	0	0	3-1-2-4-5	0	0	1	1	1	1	1	0	0	0	0		134	74	69	0	95	17	15	1	4	1	55	0		1	0		0		0		0		51	3		0	0		0	0					3		3		
1	1	1	0	0	0	0	0	0	0	0	5-8	0	0	0	0	0	0	1	0	1	0	0		114	54	58	0	100	13	15	1	4	1	50	1	47	5	1	55	0		1	19	1	8	52	2	1	0	0	0	0	0	0	0	0	1	3	2	3	3	2

Data	Details
ic1	Age over 18
ic2	Investigating center
ic3	Cardiac magnetic resonance imaging
nic1	Age under 18
nic2	No French social security scheme
nic3	Deprivation of liberty
nic4	Under legal guardianship
nic5	Patient's refusal to participate in clinical research
nic6	Impossibility of informed consent
nic7	Pregnant
nic8	Exclusion period determined by another study
med_hist	Medical history
med_hist_9	Medical history (choice=Chronic heart failure)
med_hist_6	Medical history (choice=Coronary artery disease)
med_hist_3	Medical history (choice=Diabetes-related health problems)
med_hist_1	Medical history (choice=Hypertension)
med_hist_2	Medical history (choice=Dyslipidemia)
med_hist_4	Medical history (choice=BMI > 30 kg/m)
med_hist_5	Medical history (choice=Smoking)
med_hist_7	Medical history (choice=Valvulvar disease)
med_hist_8	Medical history (choice=Heart rhythm disorders)
med_hist_99	Medical history (choice=No medical history)
previous_lvef_yn	Previously known LVEF
previous_lvef_yn	Previous LVEF
sbp	Systolic blood pressure (mmHg)
dbp	Diastolic blodd pressure (mmHg)
hr	Heart rate (bpm)
af	Atrial fibrillation
spo2	SpO2 (%)
rr	Respiratory rate (min-1)
glasg	Glasgow Coma Scale
indic_mri	Indication for cardiac MRI
pud_qual	Quality of images (pocket ultrasound devices)
pud_visual_yn	Visual estimating with PUD
pud_visual_lvef	Visual LVEF (%) (pocket ultrasound devices)
pud_auto_yn	Auto-EF with PUD
pud_auto_lvef	Auto-EF with PUD (%)
cu_qual	Quality of images (conventional ultrasound device)
cu_visual_yn	Visual estimating (conventional ultrasound device)
cu_visual_lvef	Visual LVEF (%) (conventional ultrasound device)
cu_auto_yn	Auto-EF (conventional ultrasound device)
cu_auto_lvef	Auto-EF (%) (conventional ultrasound device)
mapse_yn	MAPSE measurement
mapse	MAPSE (mm)
sa_yn	Sa measurement
sa	Sa (cm.s-1)
lvef_mri	LVEF (%) with MRI
diag_mri	Final diagnosis with MRI
pud_auto_lvef_alt	Auto-EF with PUD : LVEF < 50
pud_visual_lvef_alt	Visual LVEF with PUD : LVEF < 50
lvef_mri_alt	LVEF with MRI : LVEF < 50
pud_auto_lvef_alts	Auto-EF with PUD : LVEF < 30
pud_visual_lvef_alts	Visual LVEF with PUD : LVEF < 30
lvef_mri_alts	LVEF with MRI : LVEF < 30
mapse_inf7	MAPSE < 7
mapse_inf10	MAPSE < 10
sa_inf6	Sa < 6
sa_inf9	Sa < 9
lvef_mri_cl	LVEF with MRI
pud_auto_lvef_cl	Auto-EF with PUD
pud_visual_lvef_cl	Visual LVEF with PUD
mapse_cl	MAPSE
sa_cl	Sa
