# Supplementary material for: Evaluation of left ventricular ejection fraction by a new automatic tool on a pocket ultrasound device: Concordance study with cardiac magnetic resonance imaging
Source: PLoS One. 2024 Aug 12;19(8):e0308580. doi: 10.1371/journal.pone.0308580 (PMC11318925; doi:10.1371/journal.pone.0308580)
Supplement: Attachment — Submitted filename: Response to Reviewers.docx [file pone.0308580.s002.docx]

**Evaluation of left ventricular ejection fraction by a new automatic tool on a pocket ultrasound device: concordance study with cardiac magnetic resonance imaging**

**Auto-Ejection Fraction compared with cardiac magnetic resonance imaging**

Dear Editor:

We thank the Editor and the Referees for their very constructive comments and remarks. We hope that this new version might be found acceptable and addresses all the questions and concerns raised. As requested, we have attempted to answer to each referee comment and point raised. Moreover, our changes are distinguished by using a different font or color (red). Whatever your further answers, we are impressed by the attention you showed our work and the quality of your advice. We hope this new version will be worthy of your time and efforts.

Yours sincerely,

Thibaut Markarian, M.D.

**Journal Requirements**:

1. Please ensure that your manuscript meets PLOS ONE's style requirements, including those for file naming. The PLOS ONE style templates can be found at

https://journals.plos.org/plosone/s/file?id=wjVg/PLOSOne_formatting_sample_main_body.pdf and

https://journals.plos.org/plosone/s/file?id=ba62/PLOSOne_formatting_sample_title_authors_affiliations.pdf

An update has been provided in accordance with PLOS ONE's style requirements.

2. Thank you for submitting the above manuscript to PLOS ONE. During our internal evaluation of the manuscript, we found significant text overlap between your submission and previous work in the [introduction, conclusion, etc.].
    
   We would like to make you aware that copying extracts from previous publications, especially outside the methods section, word-for-word is unacceptable. In addition, the reproduction of text from published reports has implications for the copyright that may apply to the publications.
    
   Please revise the manuscript to rephrase the duplicated text, cite your sources, and provide details as to how the current manuscript advances on previous work. Please note that further consideration is dependent on the submission of a manuscript that addresses these concerns about the overlap in text with published work.

[If the overlap is with the authors’ own works: Moreover, upon submission, authors must confirm that the manuscript, or any related manuscript, is not currently under consideration or accepted elsewhere. If related work has been submitted to PLOS ONE or elsewhere, authors must include a copy with the submitted article. Reviewers will be asked to comment on the overlap between related submissions (http://journals.plos.org/plosone/s/submission-guidelines#loc-related-manuscripts).]

We will carefully review your manuscript upon resubmission and further consideration of the manuscript is dependent on the text overlap being addressed in full. Please ensure that your revision is thorough as failure to address the concerns to our satisfaction may result in your submission not being considered further.

We apologize for any similarities in the text with previous publications. Please be assured that there is nothing intentional. We have tried our best to correct sentences that may have been inspired by other sources. However, as we don't have any specific software at our disposal, we are willing to make any necessary changes if you let us know of any similarities.

3. Thank you for stating the following in the Competing Interests section:
   "I have read the journal's policy and the authors of this manuscript have the following competing interests: TM and XB declare a competing interest as US teachers for GE (GE MEDICAL SYSTEMS ULTRASOUND) customers. The other authors state they have no competing interests."

Please confirm that this does not alter your adherence to all PLOS ONE policies on sharing data and materials, by including the following statement: "This does not alter our adherence to  PLOS ONE policies on sharing data and materials.” (as detailed online in our guide for authors http://journals.plos.org/plosone/s/competing-interests).  If there are restrictions on sharing of data and/or materials, please state these. Please note that we cannot proceed with consideration of your article until this information has been declared.

Please include your updated Competing Interests statement in your cover letter; we will change the online submission form on your behalf.

The declaration of competing interests has been updated in the cover letter as requested.

4. In the online submission form, you indicated that [Data available on request from the corresponding author.].
   All PLOS journals now require all data underlying the findings described in their manuscript to be freely available to other researchers, either 1. In a public repository, 2. Within the manuscript itself, or 3. Uploaded as supplementary information.
   This policy applies to all data except where public deposition would breach compliance with the protocol approved by your research ethics board. If your data cannot be made publicly available for ethical or legal reasons (e.g., public availability would compromise patient privacy), please explain your reasons on resubmission and your exemption request will be escalated for approval.

We take PLOS ONE's policy into consideration and have provided all data underlying the findings described as supplementary information.

5. Please review your reference list to ensure that it is complete and correct. If you have cited papers that have been retracted, please include the rationale for doing so in the manuscript text, or remove these references and replace them with relevant current references. Any changes to the reference list should be mentioned in the rebuttal letter that accompanies your revised manuscript. If you need to cite a retracted article, indicate the article’s retracted status in the References list and also include a citation and full reference for the retraction notice.

We confirm that the references list is complete and correct. There are no retracted articles in this reference list.

**Reviewer’s comments**:

**Comments to the Author**

1. Is the manuscript technically sound, and do the data support the conclusions?

Reviewer #1: Yes

Thank you for this answer.

2. Has the statistical analysis been performed appropriately and rigorously?

Reviewer #1: Yes

Thank you for this answer.

3. Have the authors made all data underlying the findings in their manuscript fully available?

Reviewer #1: Yes

All data underlying the findings described have been added to the supplementary information.

4. Is the manuscript presented in an intelligible fashion and written in standard English?

Reviewer #1: Yes

Thank you for this answer.

5. Review Comments to the Author

   Reviewer #1: The author demonstrates the reliability of automatic evaluation of left ventricular ejection fraction (LVEF) on pocket ultrasound devices (PUD) by comparing with LVEF by cardiac magnetic resonance imaging (MRI). This is well-designed and well-written paper. The reviewer provides several suggestions.

Thank you for this comment.

1. As the author mentioned, the PUD is useful tool in emergency medicine. However, in the field of emergency medicine, the echocardiography is often performed in bad condition. Therefore, it is often impossible to obtain good quality echocardiographic images, which is expected to be a disadvantage for automatic EF evaluation. I propose to add this viewpoint in the discussion.

We agree with the reviewer's comment and have added it to the discussion.

2. The authors might suggest ways in which the accuracy of automatic EF could be improved, such as adjustment of gain and flame rate.

We totally agree with the reviewer and have added this comment to the discussion.

3. It is desirable to describe the method of LVEF assessment during atrial fibrillation in this study

We thank the reviewer for this pertinent comment. In this study, no adjustments were made for patients in atrial fibrillation. However, the Sa measurement corresponded to the mean of 3 measurements in the presence of atrial fibrillation. We have added this information in the “Ultrasound techniques” section.
